# miR-539-5p Regulates Irritable Bowel Syndrome Pathological Processes by Targeting *KDM6A*

**DOI:** 10.5152/tjg.2025.24684

**Published:** 2025-09-10

**Authors:** Yiqun Li, Zhiyu Wang, Shuangshuang Zhang, Yanling Hua, Xinting Fan, Li Li

**Affiliations:** 1 Institute of Digestive Diseases, Xuzhou Medical University, Jiangsu, China; 2 Department of Gastroenterology, The Affiliated Hospital of Xuzhou Medical University, Jiangsu, China

**Keywords:** Cell behavior, IBS, inflammatory response, *KDM6A*, LPS, miR-539-5p

## Abstract

**Background/Aims:**

The chronicity and recurrence of irritable bowel syndrome (IBS) pose significant burdens on patients’ lives, making it urgent to understand its underlying mechanisms. This study intends to investigate the function and regulatory mechanisms of miR-539-5p in IBS and lay the foundation for the creation of more effective therapeutic strategies.

**Materials and Methods:**

Rat model of IBS with diarrhea (IBS-D) was established and evaluated by the abdominal withdrawal reflex. The IBS cellular model was established in vitro using lipopolysaccharide (LPS), and real-time quantitative polymerase chain reaction was used to assess the changes in the miR-539-5p expression. Cell counting kit-8 assays, flow cytometry, and enzyme-linked immunosorbent assay were used to evaluate the effects of different treatments on cell viability, paracellular permeability, apoptosis, and inflammatory responses. Bioinformatics techniques and dual-luciferase reporter gene assays were leveraged to forecast and confirm the interaction between miR-539-5p and the *KDM6A* gene.

**Results:**

: In IBS-D rats, miR-539-5p was conspicuously downregulated, and miR-539-5p overexpression could improve the symptoms of rats. Under exposure to LPS, the expression of miR-539-5p was evidently decreased. Upregulating miR-539-5p could significantly mitigate LPS-induced cellular damage, namely inhibiting the apoptosis of intestinal mucosal epithelial cells, promoting cell proliferation, reducing paracellular permeability, and suppressing the inflammatory response. *KDM6A*, as the target gene of miR-539-5p, was remarkably upregulated in IBS-D rats and cells exposed to LPS. *KDM6A* overexpression counteracts the protective effects mediated by the upregulation of miR-539-5p.

**Conclusion:**

: miR-539-5p may be involved in regulating the pathological processes of IBS-D by targeting *KDM6A*.

**Main Points**

- miR-539-5p is downregulated in irritable bowel syndrome with diarrhea (IBS-D) rats, and its overexpression alleviates IBS-D symptoms.
- Overexpression of miR-539-5p protects against LPS-induced intestinal mucosal epithelial cell damage.
- *Kdm6a*, a target of miR-539-5p, is significantly upregulated in IBS-D rats.
- Overexpression of *KDM6A* reverses the protective effects of miR-539-5p on cells.

## Introduction

Irritable bowel syndrome (IBS) is a prevalent functional gastrointestinal disorder characterized by abdominal pain, bloating, or discomfort, often accompanied by alterations in bowel habits or stool consistency.^1^^,^^2^ Based on stool features, IBS can be further categorized into 3 subtypes: IBS-D (diarrhea-predominant), IBS-C (constipation-predominant), and IBS-M (mixed). These subtypes respectively denote patients whose primary symptoms are diarrhea, constipation, or an alternating pattern between the 2.^3^ The pathophysiology of IBS is multifaceted. It encompasses imbalances in the intestinal flora, impairments in the intestinal mucosal barrier function, low-grade intestinal inflammation, and aberrations in brain-gut axis communication. Given the varied and recurrent nature of IBS manifestations, it considerably diminishes patients’ quality of life.^4^ Currently, the precise pathogenesis of IBS remains unknown, making in-depth investigation of its molecular mechanisms crucial for overcoming research hurdles, improving understanding, and addressing the disease.

In the field of molecular biology, microRNAs (miRNAs) have been recognized as crucial regulators of various biological processes, which are also involved in disease conditions. The expression profiles of miRNAs in serum, intestinal mucosa, and other tissues of IBS patients undergo alterations, potentially reflecting the pathophysiological status of IBS.^5^^,^^6^ For instance, the expression of miR-19b in intestinal epithelial cells and its regulatory role over suppressor of cytokine signaling 3 are crucial for intestinal mucosal injury repair.^7^ A study has indicated that miR-155-5p is implicated in the pathophysiological mechanisms of IBS, and inhibiting its expression can elevate the visceral pain threshold in IBS mouse models.^8^ Zhou et al have suggested that miR-199 holds potential as a therapeutic agent for addressing visceral hyperalgesia in patients with IBS.^9^ Further investigation into the functions and regulatory mechanisms of miRNAs holds significant promise in elucidating the pathogenesis of IBS.

As an emerging miRNA molecule, miR-539-5p has attracted considerable focus due to its broad regulatory functions in key biological processes.^10^^-^^12^ Among these, its function in intestinal-related diseases is of particular interest. In colorectal cancer, miR-539-5p has been identified to exhibit a notable association with the malignant characteristics of cancer cells.^13^ Additionally, during the pathological process of pediatric pneumonia complicated by diarrhea, miR-539-5p expression undergoes marked downregulation, correlating with inflammatory responses in intestinal epithelial cells.^14^ Furthermore, in the context of inflammatory bowel diseases, miR-539-5p effectively mitigate cellular pyroptosis and inflammatory reactions by modulating the NLRP3/caspase-1 signaling pathway.^15^ However, the specific mechanistic underpinnings of miR-539-5p in IBS remain elusive.

Given the complexity of IBS and the potential role of miR-539-5p in intestinal diseases, this study investigates the regulatory mechanisms of miR-539-5p in colonic mucosal epithelial cells within the context of IBS by utilizing rat and cellular models. The objective is to gain further insights into the pathogenesis of IBS and to facilitate the development of new therapeutic targets.

## Materials and Methods

### Animals

Five specific pathogen free-grade SD pregnant rats, each weighing (360 ± 20) grams and at a gestational age of (18 ± 1) days, were purchased from the Experimental Animal Center of the Chinese Academy of Sciences (Shanghai, China). The rats were kept under optimal conditions, with indoor temperatures maintained at (22 ± 2)°C and humidity approximately 50%, ensuring ad libitum access to food and water. Following parturition, 50 pups were born, and healthy ones were selected for subsequent experimental procedures.

The study was authorized by the Xuzhou Medical University Animal Ethics Committee (No. 202503T030, Date: February 15, 2025), adhering strictly to globally accepted ethical principles and the institution’s regulations concerning animal care and experimentation. This article does not contain any studies with human subjects performed by any of the authors, informed consent statement is not required.

### Establishment of the Rat Model of Irritable Bowel Syndrome and Grouping

The objective of this study is to develop a rat model of IBS-D that more closely resembles clinical scenarios by simulating multiple etiological factors. Refer to the specific methodologies outlined in the study by Wang et al.^16^ From postnatal day 2 to day 15, pups were separated from their mothers for 3 hours daily at a fixed time, simulating the effects of early-life stress on gut development and function. At 35 days of age, pups underwent fasting with free access to water for 12 hours. Subsequently, a 1 mm diameter, single-lumen central venous catheter lubricated with paraffin oil was inserted 4 cm into the colon via the anus. A slow infusion of 1 mL of 4% acetic acid solution was administered to elicit colonic inflammation, followed immediately by tail elevation for 60 seconds to facilitate even distribution. The colon was then flushed with 1 mL of phosphate buffered saline (PBS) to mitigate residual irritation. Commencing 3 days after the acetic acid challenge (i.e., at 38 days of age), pups were subjected to chronic restraint stress. Using non-restrictive tape, their upper bodies were gently restrained to limit their ability to groom their faces with their forelimbs. This restraint lasted 2 hours daily for 7 consecutive days, simulating the effects of chronic psychological stress on gut function.

Thirty-two healthy pups were randomly and equally divided into 4 groups. The first group, designated as the control, was reared under standard conditions to ensure their health. The second group underwent the establishment of an IBS-D model, with the specific methodology detailed previously. The third group received concurrent injections of acetic acid and 0.4 mL of PBS into the peritoneal cavity, serving as the negative control (NC) for miR-539-5p upregulation, maintaining all other rearing protocols identical to those of the second group. The fourth group, alongside the acetic acid injection, administered an intra-peritoneal injection of miR-539-5p mimic (dissolved at 5 mg/kg in 0.4 mL of PBS) to augment miR-539-5p expression, with all other rearing conditions mirroring those of the second group.

### Model Evaluation and Validation

On the first day following the establishment of the model (at 45 days of age), the efficacy of modeling was validated. The rats were given free access to water and fasted for 12 hours, during which the minimum volume threshold required to elicit an abdominal withdrawal reflex (AWR) score of 3 upon colorectal balloon distension under sevoflurane anesthesia was measured and designated as the AWR minimum volume threshold. A marked decrease in this threshold indicated successful modeling. The AWR scoring system, designed to assess visceral sensitivity in rats, was evaluated by 2 independent observers using a double-blind approach. The scoring criteria were as follows: 0 points for no observable response during balloon distension, 2 points for brief head movement with subsequent cessation, 3 points for abdominal muscle contraction, and 4 points for pronounced abdominal contraction accompanied by arching of the back and pelvic elevation.^16^ Following the confirmation of successful modeling, abdominal aortic blood was collected from the rats, which were then euthanized in accordance with established protocols.

### Determination of Serum Inflammatory Factors in Rats by Enzyme-Linked Immunosorbent Assay

Blood samples were processed by centrifugation at 4°C (20 minutes) and the resulting serum (supernatant) was stored at −80°C for analysis. To quantify the levels of interleukin-1β (IL-1β), IL-6, and tumor necrosis factor-α (TNF-α) in the serum, an enzyme-linked immunosorbent assay (ELISA) assay (Jianglai Biotechnology Co., LTD, Shanghai, China) is performed following the manufacturer’s instructions. Standard solutions are diluted into 5 gradients, with 50 μL of each gradient dispensed into designated wells. The plate is incubated at 37°C for 30 minutes under a sealed plate membrane, followed by washing with PBS. After drying, equal volumes of chromogenic reagent A and reagent B are sequentially added to each well, gently mixed, and incubated at 37°C for 10 minutes in the dark for color development. After the reaction was terminated, a microplate reader (Thermo Fisher Scientific, Waltham, MA, USA) was used to detect the absorbance at 450 nm (OD_450_) and calculate the levels of inflammatory factors.

### Determination of Serum D-Lactic Acid and Diamine Oxidase in Rats by Enzyme-Linked Immunosorbent Assay

The levels of D-lactic acid (D-LA) and diamine oxidase (DAO) in rat serum were quantitatively determined using ELISA method. Adhering strictly to the detailed instructions provided in the kit (D-LA ELISA kit, and DAO ELISA kit, Jianglai Biotechnology Co., LTD, Shanghai, China), the pretreatment and dilution steps were carried out meticulously. Throughout the experimental procedure, rigorous control was exercised over the reaction conditions, encompassing temperature, duration, as well as the precise order and volume of reagent addition. Ultimately, the OD_450_ values were measured, which served as the basis for the scientific assessment of D-LA and DAO concentrations in the rat serum samples. The specific methods refer to the study by Zhu et al. ^17^ the specific methods refer to the study by Zhu et al. and the ELISA kit instructions.

### Cell Culture and Lipopolysaccharide (LPS) Induction

The NCM460 cell line, derived from human colonic mucosal epithelium, was sourced from the Cell Resource Center at the Shanghai Institutes for Biological Sciences in China. It was stored at −80°C and thawed prior to use. The cells were cultivated in Dulbecco’s modified eagle medium (DMEM) (Gibco, USA), enriched with 10% fetal bovine serum and 1% double antibiotics, maintained at 37°C, 5% CO_2_. Upon reaching approximately 80% confluence in culture dishes, subculturing was performed. The fourth generation NCM460 cells, which were in logarithmic growth, were taken and inoculated in 24-well plates. The cells were then incubated in the above incubator until they adhered to the plate surface. After attachment, the cells were exposed to varying concentrations of LPS (0, 2, 4, and 8 μg/mL, Sigma-Aldrich, St. Louis, MO, USA) for a duration of 24 hours, simulating an IBS-like environment. Refer to the specific methodologies outlined in the study by Zhang et al.^18^

### Cell Transfection

NCM460 cells treated with 8 μg/mL LPS and in good culture were screened and divided into the miR-539-5p overexpression group (miR-mimic), the miR-539-5p low expression group (miR-inhibitor) and its NC group (miR-NC), the *KDM6A* overexpression group (pcDNA3.1-*KDM6A*) and its NC group (pcDNA3.1), and a blank control group was also set up. Lipofectamine 2000 (Invitrogen, Carlsbad, USA) was utilized for transfection. Under the guidance of the instructions, the transfection reagent and plasmid were used to prepare transfection complexes and transfected into each group of cells respectively. After culturing in the incubator for 48 hours, the transfected cells that were successfully obtained were screened through real-time quantitative polymerase chain reaction (RT-qPCR) analysis.

### Real-Time Quantitative Polymerase Chain Reaction

Total RNA was extracted from rat serum and various cell groups employing the Trizol method (Tiangen Biochemical Technology Co., Ltd., Beijing, China). Specifically, 1 mL of TRIzol reagent was added to each tissue sample (weighing between 50 and 100 mg) or to cells grown in 6-well plates. These samples were then transferred to 1.5 mL Eppendorf tubes for lysis. Following lysis, 200 μL of chloroform was introduced and the mixture was centrifuged. Subsequently, the upper aqueous phase, containing the RNA, was transferred to a fresh 1.5 mL Eppendorf tube. An equal volume of isopropanol was added to this tube, and the solution was refrigerated at −20°C for 1 hour to precipitate the RNA. After rinsing with anhydrous ethanol, the RNA was dissolved in 20 μL of Rase-free double distilled water. The concentration and purity of the RNA were assessed using a Nanodrop2000 ultramicro spectrophotometer. Reverse transcription was conducted utilizing the PrimeScript® RT Master Mix Perfect Real Time Reagent Kit (Vazyme Biotech Co., Ltd., Nanjing, China). The reverse transcription system adhered to the recommended 20 μL volume (supplementary table 1), The reaction program was carried out at 37°C, 15 minutes; 85°C, 5 seconds; and 4°C, forever. The resulting cDNA was diluted 50-fold for use in RT-qPCR reactions. The RT-qPCR was executed with the RT-qPCR kit (Vazyme Biotech Co., Ltd., Nanjing, China) on a thermocycler. The specific reaction system and reaction procedure are shown in supplementary tables 2 and 3, respectively. In addition, each reaction was performed with 5 replicates. Upon completion, the relative expression levels of miRNAs (normalized to *U6*) and mRNAs (normalized to glyceraldehyde-3-phosphate dehydrogenase) were calculated using the 2^−ΔΔCt^ method. All amplification primers for this experiment were synthesized by Sangon Biotech Biological Co. (Shanghai, China), and their sequences are provided in supplementary table 4.

### Cell Proliferation

Trypsin was used to detach successfully transfected cells from the dish. The cells were subsequently transferred to a 96-well plate with a density of 1 × 10^3^ cells per well for further incubation in incubator. At the indicated time points (0, 24, 48, and 72 hours), 10 μL of cell counting kit-8 reagent (Dojindo Laboratories, Kumamoto, Japan) was introduced to each well and thoroughly mixed. Following a 4-hour period of additional incubation, allowing for the complete reaction, the optical density of each well was measured at a wavelength of 450 nm (OD_450_) using a spectrophotometer (MultiskanGo, Thermo Scientific, Waltham, MA, USA).

### Determination of Paracellular Permeability

To assess the paracellular permeability of NCM460 cells, a monolayer of intestinal epithelial barrier was established. Drawing on the research of Gregorio,^19^ Kida et al,^20^ the specific steps are as follows: NCM460 cells in control group and transfection group were carefully selected, digested, counted, and adjusted to a concentration of 1 × 10^5^ cells per milliliter. These cells were then seeded in the upper chamber of Transwell (Costar, Corning, NY, USA), with the basolateral compartment being supplemented with complete DMEM. The cells were incubated at 37°C with 5% CO_2 _until a stable monolayer barrier was achieved, confirmed by assessing transmembrane electrical resistance. Subsequently, LPS was introduced into both the apical and basolateral chambers, and the cells were further incubated for 24 hours. Then, 1 mg/mL of fluorescein isothiocyanate-dextran (FITC-dextran; Sigma-Aldrich, St. Louis, MO, USA) was introduced into the upper chamber and allowed to equilibrate for 2 hours under the same incubation conditions. Following this, 100 μL of culture medium from the lower chamber was carefully aspirated, and its OD was measured utilizing a fluorescence spectrophotometer (Hitachi, Tokyo, Japan) at excitation wavelength of 485 nm and an emission wavelength of 535 nm. Relative paracellular permeability was calculated using the method of Susan et al.^21^

### Cell Apoptosis

The cells were isolated using trypsin, followed by a wash step. After resuspension in an appropriate amount of binding buffer, 5 μL of Annexin V-FITC and Propidium Iodide (PI) were added to the cell suspension according to the requirements of the staining reagent instructions (Sobo Technology Co., Ltd., Beijing, China). Following the mixing process, the solution was incubated at normal room conditions for a period of 30 minutes, ensuring that the incubation took place in an environment devoid of light. Finally, the apoptosis rate of the cells was analyzed and assessed with a FACS Calibur flow cytometer (BD Biosciences, San Jose, USA).

### Luciferase Reporter Gene Assay

Firstly, primers were designed based on the predicted binding site to PCR-amplify 3’UTR fragments containing and lacking the miR-539-5p- and *KDM6A-*binding domain. The amplified products were then purified and tested. The purified 3’UTR fragments, both wild-type and mutant, were separately digested with enzymes and ligated into the pmirGLO dual-luciferase reporter vector. These constructs were transformed into competent cells to screen positive clones, which were further verified by colony PCR and sequencing to obtain wild-type (WT)-*KDM6A* and mutant (MUT)-*KDM6A* plasmids. According to transfection protocols, miR-mimic, miR-inhibitor, and miR-NC transfection complexes were prepared and co-transfected into NCM460 cells with either the WT-miR-539-5p or MUT-miR-539-5p plasmids. Following a 6-hour incubation period, the medium was exchanged for complete DMEM, and the cells were allowed to culture for a further 48 hours, during which cell condition was monitored and the culture environment was maintained. Following the incubation process, the cells were lysed and luciferase substrates were added to determine the relative luciferase activity.

### Bioinformatics Analysis

The downstream target genes of miR-539-5p were mined using TargetScan (https://www.targetscan.org/vert_80/), miRDB (https://mirdb.org/), and miRWalk (http://mirwalk.umm.uni-heidelberg.de/). The obtained target genes were used to draw a Venn diagram with graphing software. The functional annotations of gene ontology (GO) and Kyoto Encyclopedia of Genes and Genomes (KEGG) pathways were based on the DAVID bioinformatics tool of the National Institutes of Health (https://david.ncifcrf.gov/summary.jsp), and a visualized mRNA-protein-protein interaction (PPI) network was constructed using STRING (https://cn.string-db.org/cgi/input?sessionId=bHo5PiOTRAjM&input_page_show_search=off).

### Statistical Analysis

The data were processed and visualized using GraphPad Prism version 9.3.1, (GraphPad Software, Inc., San Diego, CA, USA) with the measurement outcomes presented as mean ± standard deviation (x ± SD). For comparisons across multiple groups, a 1-way ANOVA test was conducted, followed by Tukey’s post hoc test. Statistical significance was determined at a *P*-value threshold of < .05. 

## Results

### Construction of Irritable Bowel Syndrome with Diarrhea Rat Model

**Figure 1.**
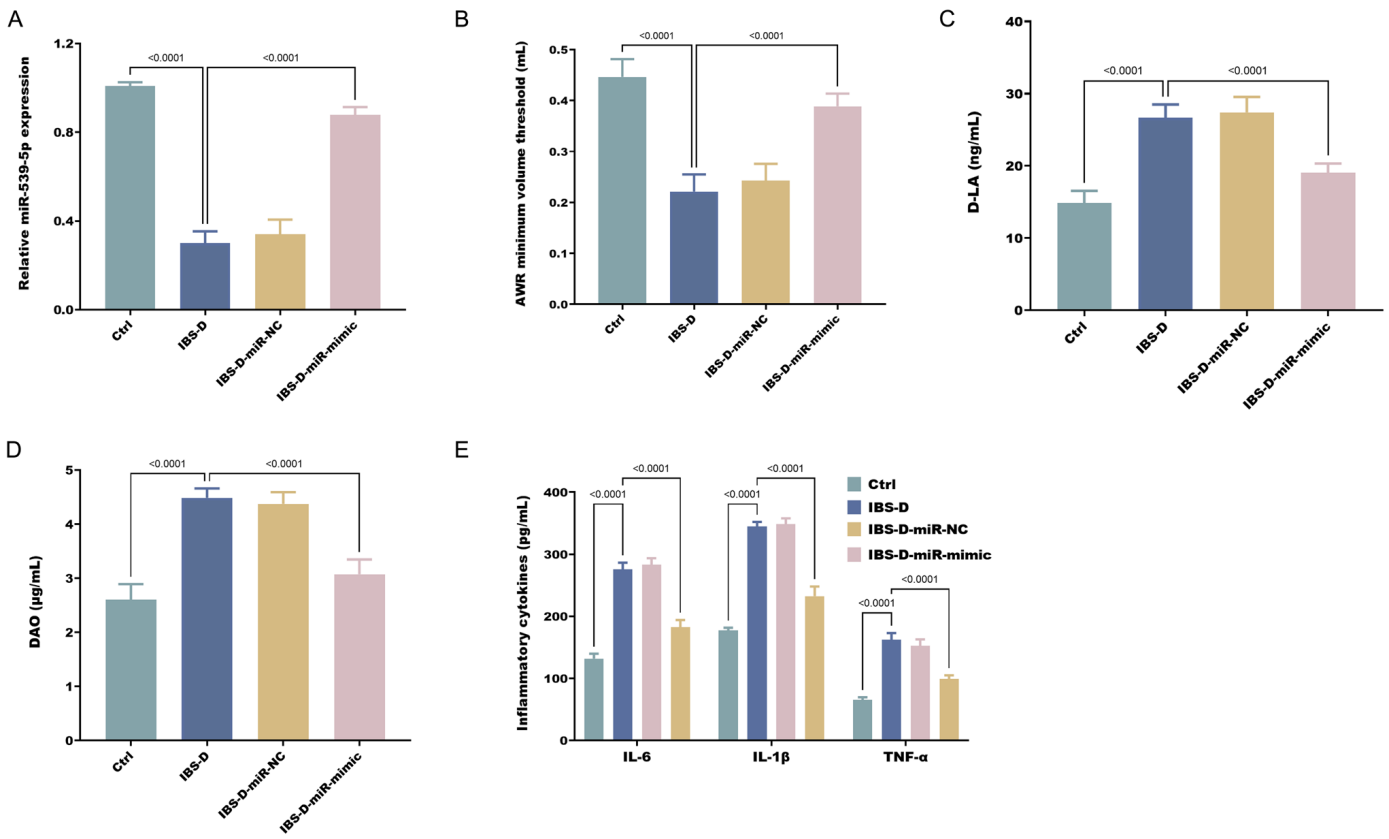
miR-539-5p is significantly downregulated in IBS-D rats (A), and its overexpression enhances AWR minimum volume threshold (B), reduces serum D-LA (C), DAO (D), and inflammatory cytokine levels (E) in IBS-D rats.

Successfully established IBS-D rat models exhibited a notable decrease in AWR minimum volume threshold, accompanied by compromised intestinal barrier function and inflammatory responses. Compared to normal rats, IBS-D rats demonstrated a significant downregulation of miR-539-5p expression (Figure 1A). However, transfection with miR-539-5p mimics caused a marked enhancement of the AWR minimum volume threshold in IBS-D rats (Figure 1B), together with suppression of D-LA levels (Figure 1C), DAO activity (Figure 1D) and pro-inflammatory cytokine production (Figure 1E).

### Construction of an Intestinal Mucosal Epithelial Cell Injury Model

**Figure 2.**
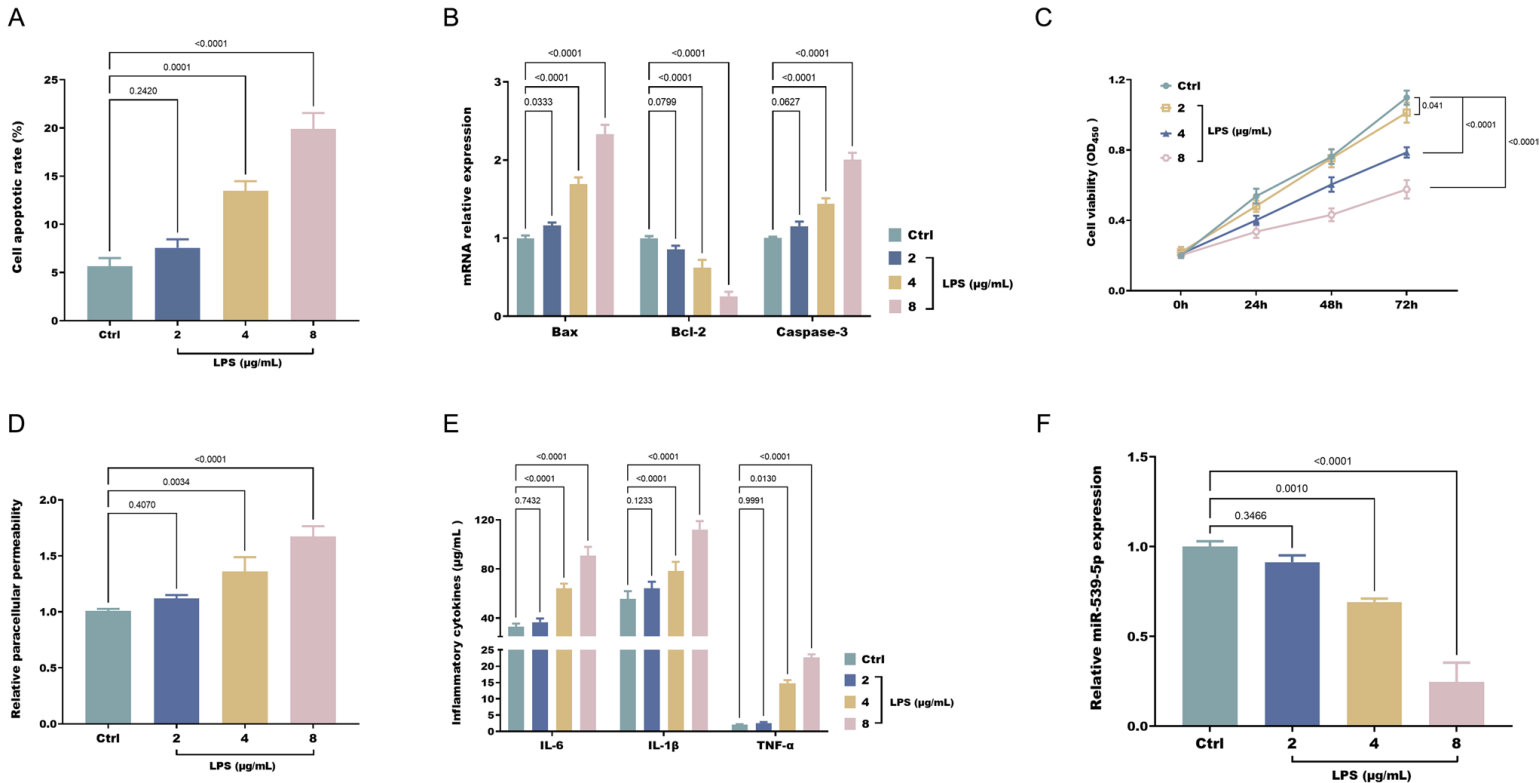
LPS induces apoptosis in NCM460 cells (A), upregulates apoptotic markers *BAX* and *CASP3* mRNA, downregulates anti-apoptotic makers *BCL2* mRNA (B), inhibits cell proliferation (C), increases paracellular permeability (D), promotes inflammation (E), and suppresses miR-539-5p expression (F).

To explore further the regulatory mechanisms of miR-539-5p, an IBS intestinal mucosal epithelial cell injury model was established using LPS. NCM460 cells were induced with varying concentrations of LPS, revealing a marked rise in the apoptotic rate of these cells as the concentration of LPS escalated (Figure 2A). This was accompanied by a notable upregulation in apoptotic markers mRNA expression (*BAX* and *CASP3*) and a downregulation of the expression of anti-apoptotic protein mRNA (*BCL2*) (Figure 2B). Additionally, the proliferative capacity of the cells decreased (Figure 2C), while paracellular permeability increased significantly (Figure 2D). The levels of pro-inflammatory cytokines in NCM460 cells also exhibited a marked elevation (Figure 2E). Notably, with increasing LPS concentration, the expression of miR-539-5p significantly was downregulated (Figure 2F).

### The Role of miR-539-5p LPS-Induced NCM460 Cell

**Figure 3.**
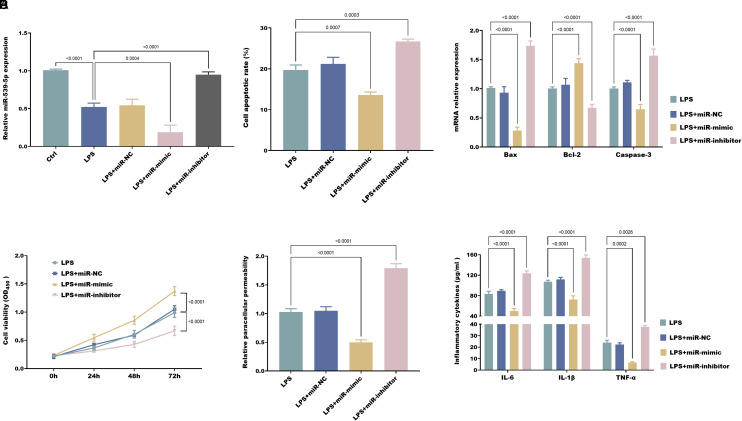
miR-539-5 p expression is modulated by transfection with miR-539-5p mimics or inhibitors (A). miR-539-5 p negatively regulates apoptosis (B-C) and positively regulates proliferation (D) in LPS-exposed NCM460 cells, while negatively regulating paracellular permeability (E) and pro-inflammatory cytokine secretion (F).

To further clarify the impact of miR-539-5p in the damage of intestinal mucosal epithelial cells induced by LPS, the expression of miR-539-5p was successfully adjusted by transfection with mimics and inhibitors (Figure 3A). In the LPS-induced cellular model, it was found that miR-539-5p overexpression reduced the apoptotic capacity of NCM460 cells (Figures 3B-C), enhanced their proliferative ability (Figure 3D), inhibited paracellular permeability (Figure 3E), and alleviated the inflammatory response exhibited by NCM460 cells (Figure 3F). Conversely, knocking down miR-539-5p expression produced the opposite effects.

### Validation of miR-539-5p Targeting *KDM6A*

To elucidate the downstream targets of miR-539-5p, an intersection analysis was conducted using the Target Scan Human, miRDB, and miRwalk datasets, resulting in the identification of 56 common target genes (supplementary figure 1A). Functional annotation through GO (supplementary figure 1B) and KEGG (supplementary figure 1C) pathways revealed that these mRNAs were predominantly enriched in intestinal function, immune response, and neuroregulation. Protein-protein interaction analysis confirmed the direct interactions among these target genes (*P* < .05), with KDM6A protein emerging as a pivotal hub within the network (supplementary figure 1D). Further investigation demonstrated that LPS stimulation induced the expression of *KDM6A* in NCM460 cells.

**Figure 4.**
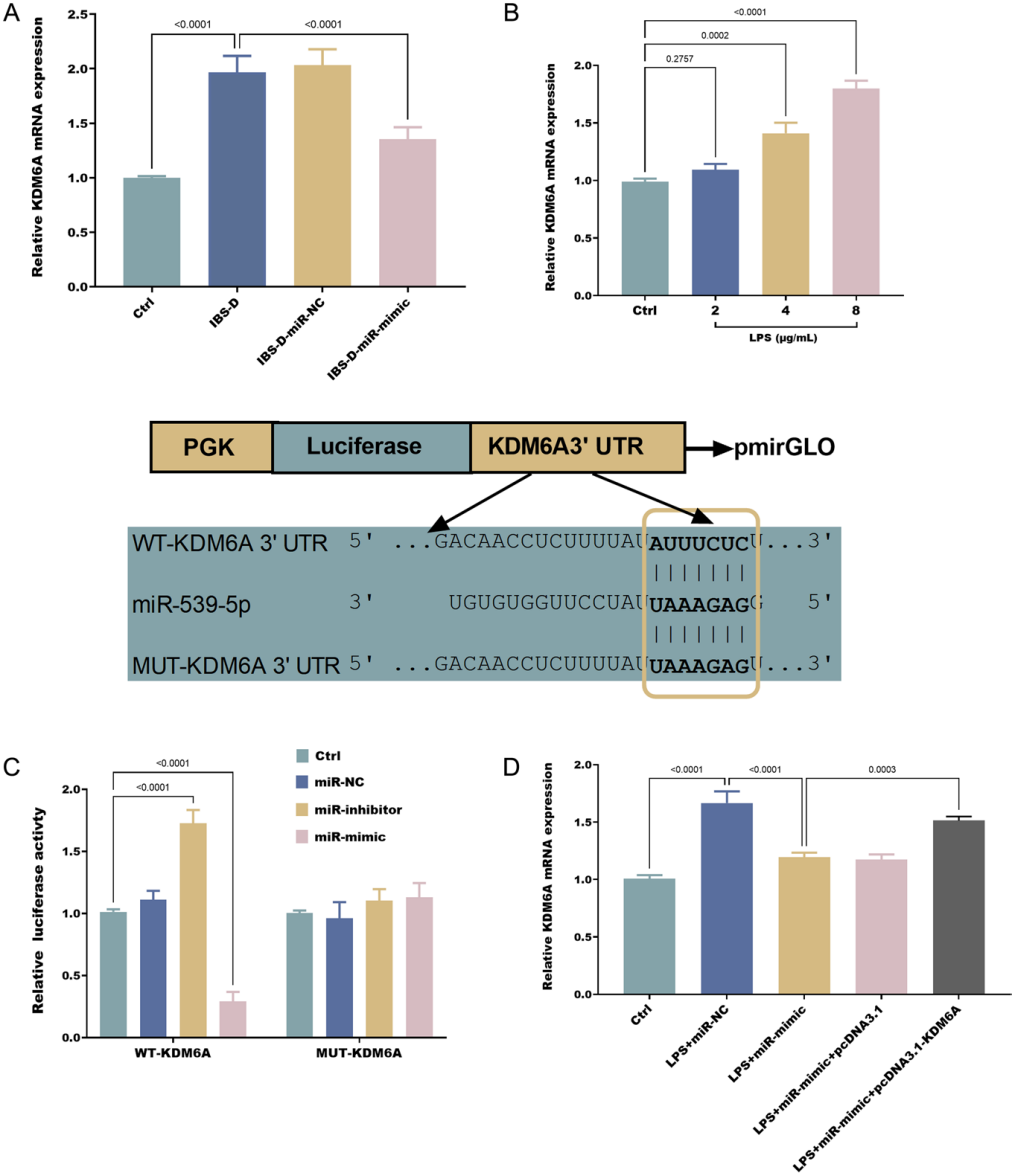
*Kdm6a* mRN A is significantly upregulated in IBS-D rats (A) and LPS-exposed NCM460 cells (B). Dual-luciferase reporter assay confirms the binding relationship between miR-539-5p and *KDM6A* in NCM460 cells (C). Overexpre ssion of miR-539-5p inhibits *KDM6A* mRNA expression in LPS-exposed NCM460 cells (D).

Additional research has shown that the expression of *Kdm6a* is markedly upregulated in IBS-D rats (Figure 4A). Furthermore, LPS stimulation can induce the expression of *KDM6A* in NCM460 cells (Figure 4B). Preliminary analysis revealed that miR-539-5p negatively regulates the relative luciferase activity of WT-*KDM6A* (Figure 4C). Moreover, in a cellular model induced by LPS, the transfection with miR-539-5p mimics led to a notable decrease in *KDM6A* expression (Figure 4D).

### The Role of *KDM6A* LPS-Induced NCM460 Cell

**Figure 5.**
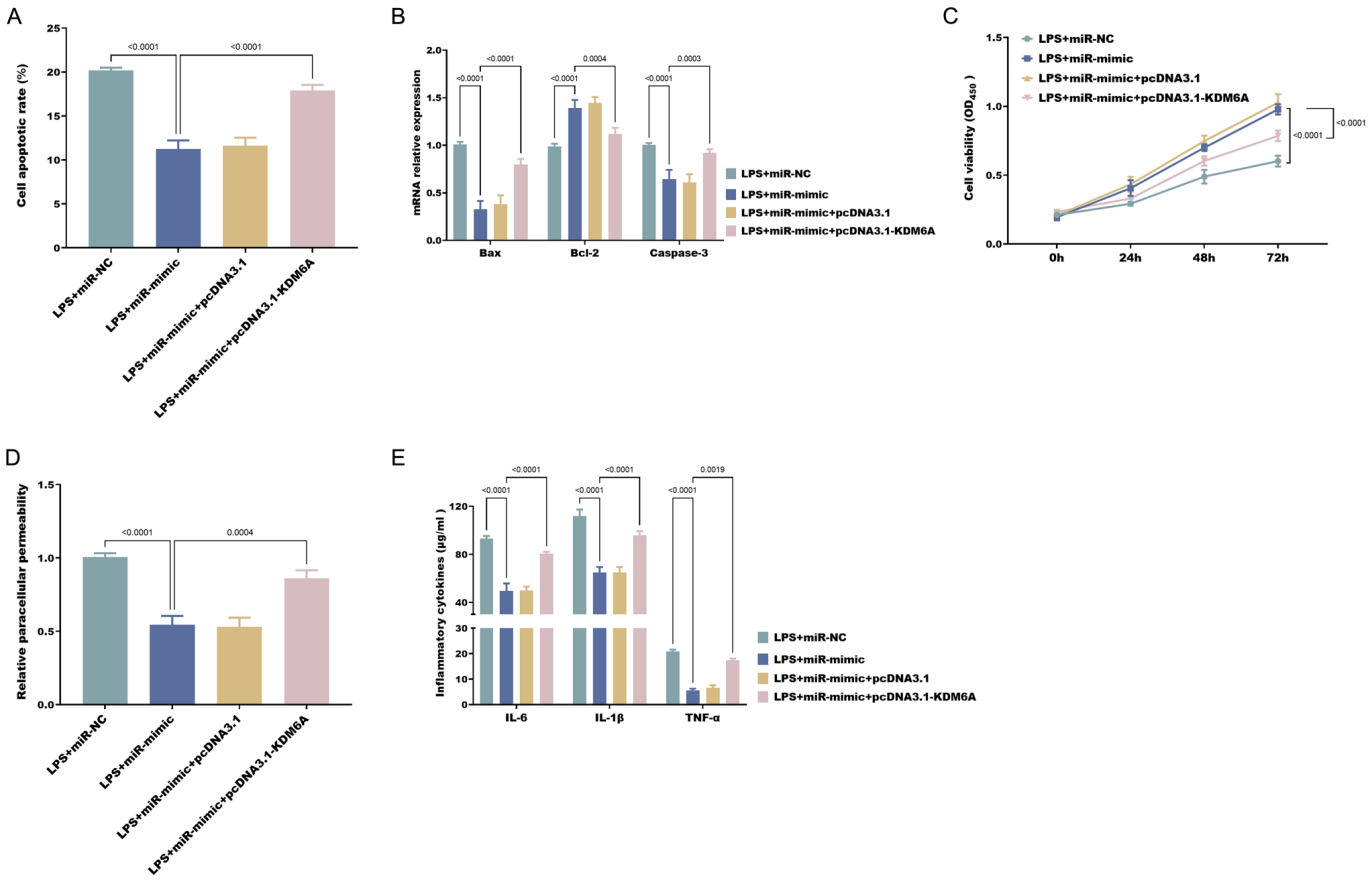
Overexpre ssion of *KDM6A* significantly promotes apoptosis (A-B), inhibits proliferation (C), increases paracellular permeability (D), and promotes pro-inflammatory cytokine secretion (E) in LPS-exposed cells.

In the LPS-induced cellular model, overexpression of *KDM6A* significantly reversed the positive effects elicited by miR-539-5p upregulation in NCM460 cells. Specifically, *KDM6A* upregulation promoted apoptotic capacity (Figure 5A-B), inhibited cell viability (Figure 5C), increased paracellular permeability (Figure 5D), and exacerbated inflammatory responses (Figure 5E) in NCM460 cells.

## Discussion

Irritable bowel syndrome, a prevalent chronic functional gastrointestinal disorder, exhibits a complex and yet incompletely elucidated pathogenesis. In recent years, the role of miRNAs in regulating gene expression, cellular functions, and pathophysiological processes has garnered increasing attention.^22^^,^^23^ This study aimed to initially investigate the role of miR-539-5p in the pathophysiology of IBS-D. An IBS-D mouse model was established as an experimental platform, with the AWR assessment system systematically introduced as a pivotal indicator of intestinal sensitivity.^24^ Research indicates that D-LA is a metabolite generated by intestinal bacteria. In the event of intestinal mucosal injury, significant amounts of D-LA can permeate into the bloodstream.^25^ Meanwhile, DAO represents an active enzyme that resides within intestinal mucosal cells. When these cells suffer damage, DAO is released into the circulatory system.^26^ Elevated levels of both D-LA and DAO display a close correlation with the extent of intestinal mucosal damage as well as changes in permeability.^27^ Furthermore, abnormal elevations in inflammatory cytokines such as IL-6, IL-1β, and TNF-α are also closely associated with significant impairment of intestinal barrier function.^28^ Crucially, the study found miR-539-5p expression to be significantly downregulated in model mice. Furthermore, miR-539-5p overexpression significantly improved AWR minimum volume threshold, reduced D-LA and DAO levels, and alleviated intestinal inflammatory responses. These findings suggest that miR-539-5p has a crucial regulatory role in the pathogenesis of IBS-D.

To preliminary investigate the specific regulatory mechanisms of miR-539-5p, an IBS cellular model was established by treating intestinal mucosal epithelial cells with exogenous LPS. Previous studies have confirmed that LPS treatment significantly inhibits intestinal mucosal epithelial cell viability, enhances paracellular permeability, and promotes cell apoptosis and inflammation.^29^ Similarly, these phenomena were also observed in this study. Notably, following LPS treatment, the expression of miR-539-5p was markedly downregulated. This discovery indicates that miR-539-5p could have a function in the process involving LPS-induced damage to intestinal mucosal epithelial cells. The results of this study demonstrate that the promotion of miR-539-5p expression can markedly alleviate the impact of LPS on the behavioral activities of intestinal mucosal epithelial cells. including restoring cell viability, reducing paracellular permeability, and decreasing cell apoptosis. Research has pointed out that the expression of miR-539-5p is markedly elevated in colorectal cancer tissues, and inhibiting its expression can restrain the proliferation and migration abilities of colorectal cancer cells and simultaneously induce apoptosis.^30^ Similarly, miR-539-5p has been discovered to regulate the proliferation and migration in vascular smooth muscle cells stimulated by ox-LDL by targeting SPP1.^12^^,^^31^ These studies provide further evidence to support the assertion that miR-539-5p plays a pivotal role in the behavioral activities of intestinal mucosal epithelial cells. Moreover, this study also discovered that miR-539-5p can negatively regulate the inflammatory response of LPS-induced intestinal mucosal epithelial cells. In the acute lung injury model, overexpression of miR-539-5p can mitigate the inflammatory response of LPS-induced pulmonary microvascular endothelial cells.^32^ In the sepsis cell model, miR-539-5p has also been found to regulate the levels of TNF-α and IL-1β in cardiomyocytes.^33^ The collective evidence indicates that miR-539-5p may act as a responsive molecule under LPS stimulation and participate in the regulation of the cellular inflammatory response process.

It was determined that the *KDM6A* serves as a target of miR-539-5p and was notably upregulated in IBS-D mice. *KDM6A*, a cell adhesion molecule, is vital for maintaining intercellular connections and barrier functions.^34^^,^^35^ The findings of this research indicate that the promotion of *KDM6A* expression may serve to reverse the protective effect of miR-539-5p upregulation on intestinal mucosal epithelial cells. Similarly, in the spinal cord injury model, the high expression of *KDM6A* coexisted with the damaged response of nerve cells, and miR-145 exhibited a neuroprotective effect by regulating the expression of *KDM6A*.^36^ Additionally, study on type 1 diabetic nephropathy have shown a correlation between elevated *KDM6A* levels and worsened renal tubular dysfunction and tissue damage, with miR-199b-3p downregulation accelerating the epithelial-to-mesenchymal transition process by influencing *KDM6A* and E-cadherin expression.^37^ The above shows that *KDM6A*, as a key node in the microRNA regulatory network, exerts a significant influence on the regulation of cell fate and tissue repair.

While this study offers initial insights into the role of miR-539-5p and *KDM6A* in IBS, it has several limitations. Firstly, immunohistochemical analysis of tight junction markers to assess intestinal permeability was not conducted. Such analysis could have provided a more detailed and direct visualization of changes in intestinal barrier function, strengthening the evidence for a connection between miR-539-5p and intestinal mucosal integrity. Additionally, further analyses of the signaling pathways involved have not been conducted. Lastly, feces samples could have provided valuable information regarding changes in the gut microbiota and intestinal permeability markers, further enriching our understanding of the role of miR-539-5p in IBS. The feces of all IBS-D rats included in this study exhibited typical diarrhea manifestations, such as pasty, loose, or watery consistency. However, difficulties were encountered when attempting to collect samples from these diarrhea rat models. The inherent complexity of feces sample collection, coupled with its susceptibility to contamination, resulted in inconsistent and unsatisfactory outcomes. Future research should address these limitations to fully elucidate the role of miR-539-5p in IBS.

In summary, miR-539-5p likely impacts the function of intestinal mucosal epithelial cells by regulating downstream target genes such as *KDM6A*, thereby participating in the onset and progression of IBS. Future research can delve deeper into the interaction mechanisms between miR-539-5p and *KDM6A*, as well as their potential therapeutic applications in IBS.

## Supplementary Materials

Supplementary Material

## Data Availability

The data that support the findings of this study are available on request from the corresponding author.
