# Supplementary material for: miR-539-5p Regulates Irritable Bowel Syndrome Pathological Processes by Targeting KDM6A
Source: Turk J Gastroenterol. 2025 Sep 10;37(1):15–25. doi: 10.5152/tjg.2025.24684 (PMC12824890; doi:10.5152/tjg.2025.24684)
Supplement: Supplementary Material [file supplementary_material.pdf]

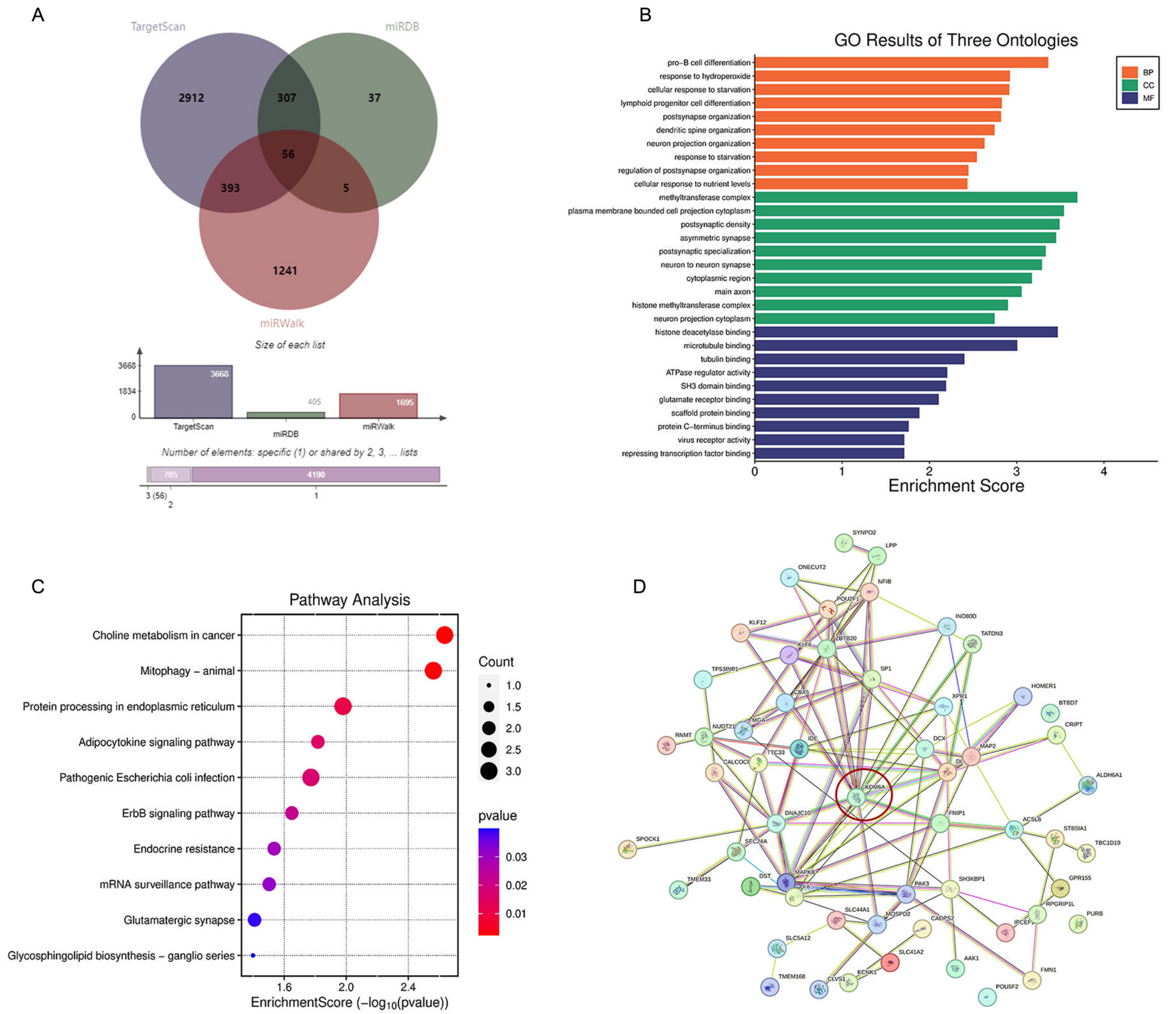

Supplementary Figure 1. A Total of 56 miR-539-5p target genes were obtained by intersecting 3 databases. GO (B) and KEGG (C) perform functional enrichment analysis on target genes. PPI analysis of target genes (D).

Supplementary Table 1. Reverse reaction system (20μL system)

Name	Dosage
Total RNA (DNA removal)	2 μg
Prime Script RT Enzyme Mix I	1 μL
Random 6 mers (100μM)	1 μL
Oligo dT Primer (50μM)	1 μL
5×Prime Script Buffer 2	4 μL
Rnase Free dH_2O	11 μL

Supplementary Table 2. RT-qPCR reaction system (20μl system)

Name	Dosage
2×SYBR Premix Ex Taq II (Tli Rnase Plus)	10 μL
Forward primer (10uM)	0.8 μL
Reverse primer (10uM)	0.8 μL
cDNA (5uM)	2 μL
Rnase Free dH_2O	6.4 μL

Supplementary Table 3. The reaction condition of RT-qPCR

Steps	Temperature	Time	Cycle
Pre-denaturation	95°C	30s	1
Denaturation	95°C	5s	40
Annealing	60°C	30	
Extension	72°C	30	1

Supplementary Table 4. Primer sequences

Primer Name		Primer sequence
KDM6A	Forward	5′-TTTGGTCTACTTCCATTACAATGCA-3'
	Reverse	5′-AAGCCCAAGTCGTAAATGAATTTC-3'
Bax	Forward	5′ CGGCGAATTGGAGATGAACTGG3′
	Reverse	5′CTGCAGAGGATGATTGCTGA3′
Caspase-3	Forward	5′-GCTATTGTGAGGCGGTTGT-3′
	Reverse	5′-TGTTTCCCTGAGGTTTGC-3′
Bcl-2	Forward	5′-TACCGTCGTGACTTCGCAGAGAT-3′
	Reverse	5′-AGGAGAAATCAAACAGAGGTCGC-3′
GAPDH	Forward	5′-GGGCATCTTGGGCTACAC-3′
	Reverse	5′-GGTCCAGGGTTTCTTACTCC-3′
miR-539-5p	Forward	5′-CCAAAGGAGCATCAGA GCAGA-3′
	Reverse	5′-AAGGGCTCGACAGAATTGGG-3′
U6	Forward	5′-CTCGCTTCGGCAGCACA-3
	Reverse	5′-AACGCTTCACGAATTTGCGT-3′

KDM6A: Lysine Demethylase 6A; Bcl-2: B-cell lymphoma-2; Bax: BCL-2-associated X protein;
